# A Medical Iconoclast

**Published:** 1886-02

**Authors:** 


### A MEDICAL ICONOCLAST.

IT will surprise many of our practicing physicians to learn, on excellent authority, that they have been for many years using certain very popular drugs on an altogether erroneous belief in their effects. We refer to the Chlorate of Potassium as a "blood oxydiser," and the Bromide of Potassium, as an agent "to diminish the amount of blood circulating in the brain"—i. e., as a cerebral depressant or sedative! We copy the following from an editorial in the *Therapeutic Gazette* for January, written, we suppose, by Dr. H. C. Wood, the Editor, or by Dr. Robert Meade Smith, the Associate Editor, both of whom are good authority:

"Possibly scores of children, certainly many lives, have been sacrificed in the last ten or fifteen years to the theory that the chlorate of potassium yields its oxygen in the blood, and thereby purifies the vital fluid; a theory as baseless as it is ingenious and attractive, but which still has its advocates, and which, within a few weeks, we saw taught by a learned professor to a gaping crowd of students—a theory which led in practice to the immoderate use of the chlorate in diphtheria, and no doubt to a notable increase in the death roll.

"Another theory almost as universally believed in, and almost as groundless as the one just spoken of, is that which attributes the effects of bromide of potassium upon the nervous system to contraction of the finer blood-vessels, and the consequent production of cerebral and spinal anæmia. How many pages have been written in regard to this theory! What great discourses and imaginings concerning the nature of epilepsy and various other diseases, we have built upon it! How it still permeates neurological writings! and yet there never has been a single well observed fact supporting it. Evolved out of the inner consciousness of the neurological clinician, it has been swallowed by a profession whose gullibility is still marvelous and triumphant."

There! thus at one cruel kick Dr. Wood has demolished one of the prettiest little crystal palaces,—one of the lovliest and most fascinating little graven images to which the medical profession ever bowed down and worshipped! The theory of imparting oxygen to the blood by the use of the chlorate and chlorides has been the basis on which the treatment of a very great many diseases—especially those we call "malignant"—has been constructed. Watson's celebrated "chlorine mixture," for scarlet fever, and Fenner's adaptation of it to yellow fever, and later, Dr. Wood's own (if we are not greatly mistaken) quite celebrated prescription of the chlorate of potassium, chlorinated tincture of iron, and quinine, for diphtheria, are all founded on the fascinating idea of "oxydising the blood." There, now! gone glimmering! gone, as the wag said, "where the woodchuck chucketh," and nothing left in its place!—no substitute, no suggestion left, what to do instead! Now, when we encounter a case of any of the aforesaid diseases, we will feel like the boy did, when he found he had forgotten to load his gun,—only when he came up on the ducks,—got nothing to shoot 'em with. Really, Dr. Wood, you do not know the extent of damage you have done the profession by thus knocking down their "image,"--however many lives you may *think* you have saved by disarming the M. Ds. of the terrible chlorate and bromide. In other words—if chlorate potassa is not "good for" diphtheria, putrid sore throat, etc., what is? That's what the average practitioner will want to know.

